# Isolation of Genetically Diverse H5N8 Avian Influenza Viruses in Poultry in Egypt, 2019–2021

**DOI:** 10.3390/v14071431

**Published:** 2022-06-29

**Authors:** Ahmed H. Salaheldin, Ahmed R. Elbestawy, Abdelkader M. Abdelkader, Hesham A. Sultan, Awad A. Ibrahim, Hatem S. Abd El-Hamid, Elsayed M. Abdelwhab

**Affiliations:** 1 Department Poultry and Fish Diseases, Faculty of Veterinary Medicine, Alexandria University, El-Beheira 22758, Egypt; 2 Department of Poultry and Fish Diseases, Faculty of Veterinary Medicine, Damanhour University, Damanhour 22511, Egypt; ahmed.elbestawy@vetmed.dmu.edu.eg (A.R.E.); drhatem_deltavet@yahoo.co.uk (H.S.A.E.-H.); 3 Faculty of Veterinary Medicine, Damanhour University, Damanhour 22511, Egypt; Abdelkadermohammed37@yahoo.com; 4 Department of Poultry and Rabbit Diseases, Faculty of Veterinary Medicine, University of Sadat City, Menoufiya 32958, Egypt; hisham.sultan@vet.usc.edu.eg; 5 Department of Avian and Rabbit Medicine, Faculty of Veterinary Medicine, Assiut University, Assiut 710526, Egypt; awadfadel@yahoo.com; 6 Institute of Molecular Virology and Cell Biology, Federal Research Institute for Animal Health, Friedrich-Loeffler-Institut, Insel Riems, 17493 Greifswald, Germany

**Keywords:** avian influenza virus, H5N8, clade 2.3.4.4b, Egypt, poultry, vaccination failure

## Abstract

The global spread of avian influenza virus (AIV) of clade 2.3.4.4b since 2016 has caused severe losses in wild birds and poultry and has posed a risk for the infection of mammals including humans. The vaccination of poultry has been used to limit the spread of the virus and mitigate its socioeconomic impact. Here, we describe H5N8 epidemics in chickens, turkeys and ducks from different localities in Egypt from 2019 to 2021. About 41.7% (*n* = 88/211) flocks were tested positive by RT-qPCR for H5N8 viruses with prevalence rates of 45.1% (*n* = 65/144) and 34.3% (*n* = 23/67) in vaccinated and non-vaccinated flocks, respectively. A sequence analysis of the hemagglutinin and neuraminidase genes indicated not only the multiple introduction events of H5N8 viruses in Egypt but also the establishment of endemic viruses in commercial poultry in 2020/2021. The recent H5N8 viruses in poultry in Egypt are genetically distinct from the majority of licensed vaccines used in the field. Together, our findings indicate that poultry in Egypt is an endemic center for clade 2.3.4.4b in the Middle East. The efficiency of current vaccines should be regularly evaluated and updated to fully protect poultry flocks in Egypt against H5N8 viruses.

## 1. Introduction

Avian influenza viruses (AIV) belong to the family *Orthomyxoviridae* and infect a wide range of avian and mammalian species [1]. They are classified according to the antigenic properties of the surface glycoproteins hemagglutinin (HA) and neuraminidase (NA) into 16 HA and 9 NA subtypes, respectively. All AIV subtypes were isolated from wild birds, the natural reservoir, where the infection is usually asymptomatic, with few exceptions. In domestic birds, all AIVs are low pathogenic (LP), while some H5 and H7 subtypes can be highly pathogenic (HP) [2]. Since 1996, the HPAIV H5 subtype of the Goose/Guangdong lineage continues to cause severe economic losses in the poultry industry and pose a significant pandemic threat. The virus has been diversified into 10 HA phylogenetic clades (clades 0 to 9) and tens of suborder clades [3]. In 2014, clade 2.3.4.4a spread from Asia to Europe and North America, while in 2016 clade 2.3.4.4b spread from Asia to Europe and Africa [4,5]. In addition to the high mortality in domestic and wild birds, the virus succeeded to jump species barriers and infected mammals including humans, foxes and seals in several countries [6,7,8,9]. Therefore, it is paramount to efficiently control the virus in poultry to limit bird-to-human transmission [10].

In addition to the biosecurity measures and culling strategy, the mass vaccination of poultry is highly useful to protect poultry from AIV and prevent spillover to other mammals including humans [11]. Several AIV vaccines have been developed including inactivated whole virus vaccines and recombinant virus vector vaccines [12]. Experimental and field studies showed that the use of H5 or H7 vaccines, particularly those containing antigenically matched hemagglutinin similar to the field viruses, were effective at preventing morbidity and mortality in poultry, limiting virus replication, reducing viral loads in the environment, and interrupting poultry-to-poultry transmission [11,13,14,15]. Importantly, the effective vaccination of poultry against AIV (e.g., H7N9 in China) successfully eliminated human infection, emphasizing the importance of the active control of animal diseases in the one-health concept [16]. Similar to human influenza viruses, AIV vaccines should be regularly updated to fully protect poultry against exotic and newly introduced subtypes/serotypes [11,15]. The best example of the regular updating of AIV vaccine strains is China, where several AIV subtypes including H5/H7 AIV are endemic in poultry. Recently, an updated trivalent vaccine (H5-Re13, H5-Re14, and H7-Re4, of which the HA and NA genes originated from the newly detected H5N6 virus, H5N8 virus, and H7N9 virus, respectively) has been developed. Animal studies proved that the novel H5/H7 trivalent vaccine is immunogenic and could provide solid protection against viruses that are currently circulating in nature [17]. Outside China (e.g., in Egypt and Mexico), the use of outdated vaccines or mismatched vaccine strains conferred suboptimal protection to poultry against HPAIV [18,19].

In Egypt, three zoonotic AIVs have been detected in poultry including H5N1 (2005–2020), H9N2 (since 2013) and recently H5N8 clade 2.3.4.4b (since 2016) [20,21]. The latter virus was transmitted from wild birds, spread widely in commercial farms and replaced the endemic H5N1 in poultry [22]. Sequence analyses of H5N8 clade 2.3.4.4B in poultry in Egypt from 2016 to 2018 revealed multiple introductions via migratory birds along the Black Sea–Mediterranean and East African–West Asian migration flyways [23,24,25]. The location of Egypt on these two intersecting flyways of migratory birds from Asia and Europe represents a major threat for the introduction of AIV in poultry and its spread to neighboring countries [26,27]. It is worth mentioning that Egypt is the country with the highest confirmed human H5N1 infections worldwide [28], and clinical and subclinical H9N2 infections in humans have been reported [29]. Therefore, it is important to understand the epidemiology and evolution of these zoonotic AIVs in Egyptian poultry [20]. To mitigate the socioeconomic losses in the poultry industry, Egypt mainly embarked on the use of blanket H5/H9 vaccination campaigns, particularly in the commercial sector [30,31]. Nevertheless, H5N1 and to a lesser extent H9N2 viruses have been frequently isolated in vaccinated flocks [31]. The upsurge of H5N1 in vaccinated flocks in Egypt was associated with the evolution of antigenic drift variants, which acquired several mutations in the HA immunogenic epitopes under vaccination pressure, likely due to the use of antigenically mismatched vaccine strains [32,33]. For the recent H5N8 viruses, the prevalence of this virus in vaccinated flocks is not fully understood.

In this study, swab samples were collected from commercial chickens, ducks and turkey flocks with a history of respiratory signs and high morbidity and mortality rates. Virus detection, isolation and sequencing of the HA and NA of selected samples were performed. To understand the molecular epidemiology of H5N8 viruses in Egypt, we analyzed the HA/NA sequences from all H5N8 viruses in the GISAID from Eurasia and the Middle East since 2016.

## 2. Materials and Methods

### 2.1. Virus Detection and Isolation

Tracheal and cloacal swabs were collected and submitted to the laboratory between November 2019 to March 2021. Samples (10 to 20 swabs from each flock) were collected from commercial poultry farms in different provinces in Egypt. Pooled samples were tested using RT-qPCR targeting the M gene of influenza A virus [34] and positive samples were further tested to detect the HA and NA subtypes [34] using a multiplex Real-Time RT-PCR applied biosystem 7500. Positive samples were inoculated in 10–12 day-old embryonated chicken eggs via the allantoic sac for one or two passages in a Class III Biosafety Cabinet. Allantoic fluids were tested using the hemagglutination test according to the OIE protocol.

### 2.2. Sequence of the HA and NA Genes

The HA and NA genes of the selected samples were amplified using one-step conventional RT-PCR according to the manufacturer instructions (SuperScript™ III One-Step RT-PCR System with Platinum™ Taq DNA Polymerase, Thermofisher, Waltham, MA, USA). Samples were purified from 1.5% gel using QIAquick Gel Extraction Kit (Qiagen, Hilden, Germany). Purified segments were subjected to sequencing using BigDye Terminator v3.1 Cycle Sequencing Kit (Applied Biosystems, Waltham, MA, USA) and universal forward and reverse primers as previously published [35,36]. Sequences were assembled and edited using Geneious Prime® 2021.0.1. We further compared the amino acid (aa) differences in HA of Egyptian H5N8 viruses, including the new sequences generated in this study as well as the European viruses from 2017 to 2021, to the sequence of the commercially available H5 vaccines in poultry in Egypt.

### 2.3. Phylogenetic Analysis

Nucleotide sequences of all-full or near-full HA (*n* = 2700) and NA (*n* = 1994) genes of H5N8 viruses from Asia, Africa, the Middle East (i.e., Iran, Iraq, Israel, Saudi Arabia) and Europe after a BLAST search in GISAID were downloaded. The date of data retrieval was 21 January 2022. The sequences, including 20 new HA and 17 new NA sequences generated in this study, were aligned using a multiple sequence alignment program (MAFFT). We first generated HA and NA phylogenetic trees for all HA/NA sequences by IQTree [37] and MrBayes implemented in Topali v2 [38]. Thereafter, MrBayes was used to determine the best nucleotide substitution model. Trees were generated after selecting 4 chains of 10,000,000 replicates and 25% buried-in parameters. Posterior probability values are shown on the main nodes. Deduced amino acid (aa) sequences were analyzed by Geneious Prime® 2021.0.1.

## 3. Results

### 3.1. Surveillance

The samples in this passive surveillance were submitted from flocks suffering from respiratory disorders and elevated morbidity or mortality and/or a reduction in egg production in layers’ and breeders’ birds. Samples from 211 commercial poultry flocks representing 67 non-vaccinated and 144 vaccinated flocks from different localities in Egypt were examined. About 41.7% (*n* = 88/211) of flocks were tested positive by RT-qPCR for H5N8 viruses. The prevalence in vaccinated flocks was higher than in non-vaccinated flocks, where 45.1% (*n* = 65/144) and 34.3% (*n* = 23/67) were positive, respectively. Epidemiological data for selected outbreaks are summarized in Table 1. We successfully isolated 20 viruses in embryonated chicken eggs and amplified the NA and/or HA by RT-PCR and subjected them to sequencing. These samples represented 15 chicken flocks (broiler, layers, breeders) with a capacity ranging from 500 to 35,000 chickens, 2 duck flocks with a capacity of 10,000 to 12,000 and 3 turkey flocks with a capacity of 5000 to 7000 turkeys. They were collected and submitted to the laboratory in 2019 (*n* = 8), 2020 (*n* = 10) and 2021 (*n* = 2). The age of the chickens ranged from 19 days to 40 weeks, ducks from 40 to 45 days and turkeys from 55 to 90 days. All flocks were vaccinated at least once except for three broiler chicken flocks that were not vaccinated. Two layer chicken and one broiler breeder flocks were vaccinated three and four times, respectively (Table 1). The sequences were submitted to the GISAID and assigned accession numbers EPI1999273 to EPI1999312 (Table 1).

### 3.2. HA Phylogenetic Analysis

**Figure 1:**
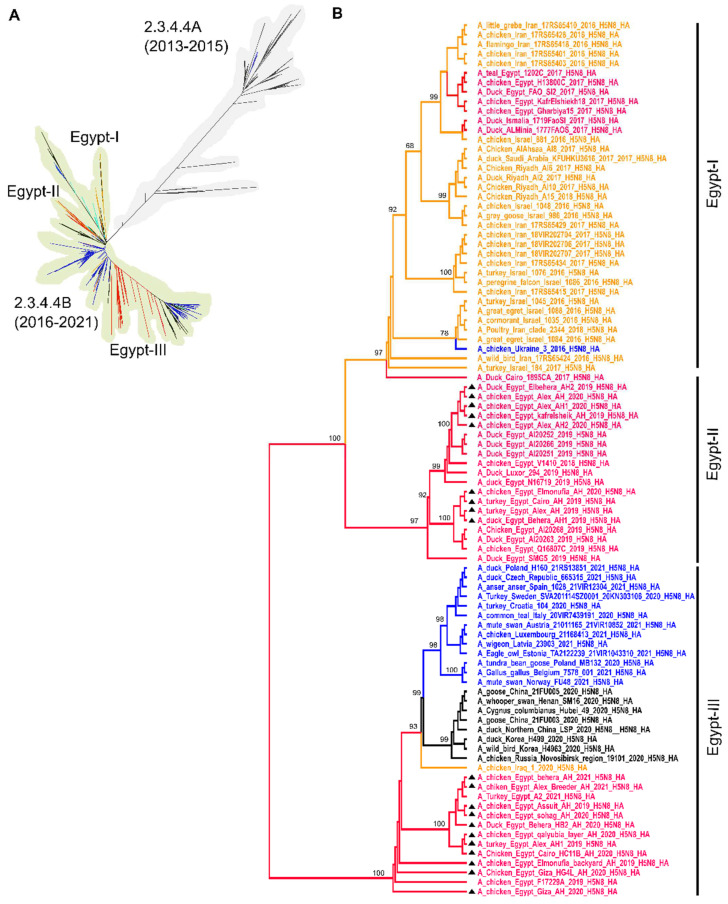
Phylogenetic relatedness of the HA of Egyptian H5N8 to Eurasian viruses. Nucleotide sequences of all-full or near-full HA (*n* = 2700) genes of H5N8 viruses from Asia, Africa, the Middle East and Europe were retrieved on 21 January 2022 and aligned using MAFFT, and the tree was generated by IQTree. The tree shows clade 2.3.4.4a mainly circulated in wild birds and rarely in poultry from 2013 to 2015, and clade 2.3.4.4B viruses from 2016 to 2021 (**A**). The phylogenetic tree of the Egyptian H5N8 viruses in clade 2.3.4.4B, including new sequences generated in this study (marked in black triangles), and selected sequences from other countries was generated by MrBayes implemented in Topali v.2 using the GTR + G model. Trees were generated after selecting 4 chains of 10,000,000 replicates and 25% buried-in parameters. Posterior probability values are shown on the main nodes (**B**). Egyptian H5N8 sequences are shown in red, H5N8 sequences from the Middle East are shown in orange, sequences from Europe are shown in blue and Asian viruses are in black. Branches depicted in cyan in panel A refer to sequences from other African countries.

The analyzed sequences of the HA from Egypt represented viruses from 2016 to 2021 including twenty new HA sequences in this study. The phylogenetic analysis indicated that the Egyptian viruses of domestic birds are allocated in three major phylogroups (Figure 1A), and a few Egyptian viruses from wild birds from 2016 (*n* = 6) are sporadically found in different groups. Phylogroup-I and II are two daughter clusters and distinct from phylogroup-III. Phylogroup-I includes a large proportion of H5N8 viruses from wild and domestic birds in Egypt and the Middle East (i.e., Iran, Israel and Saudi Arabia) in 2016–2018. This group indicated the parallel and multiple dispersals of diverse H5N8 viruses in the Middle East and Africa in 2016–2018. They were not detected after 2018. Phylogroup-II contains only Egyptian viruses, including nine new sequences generated in this study, from domestic birds (i.e., chickens, ducks and turkeys) from 2017 to 2020. Phylogroup-III contains viruses from poultry in Egypt from 2017 to 2021 including 11 new sequences generated in this study as well as published sequences from Europe, Iraq, the Russian Federation and Asia (in 2020 and 2021) (Figure 1B).

### 3.3. NA Phylogenetic Analysis

**Figure 2:**
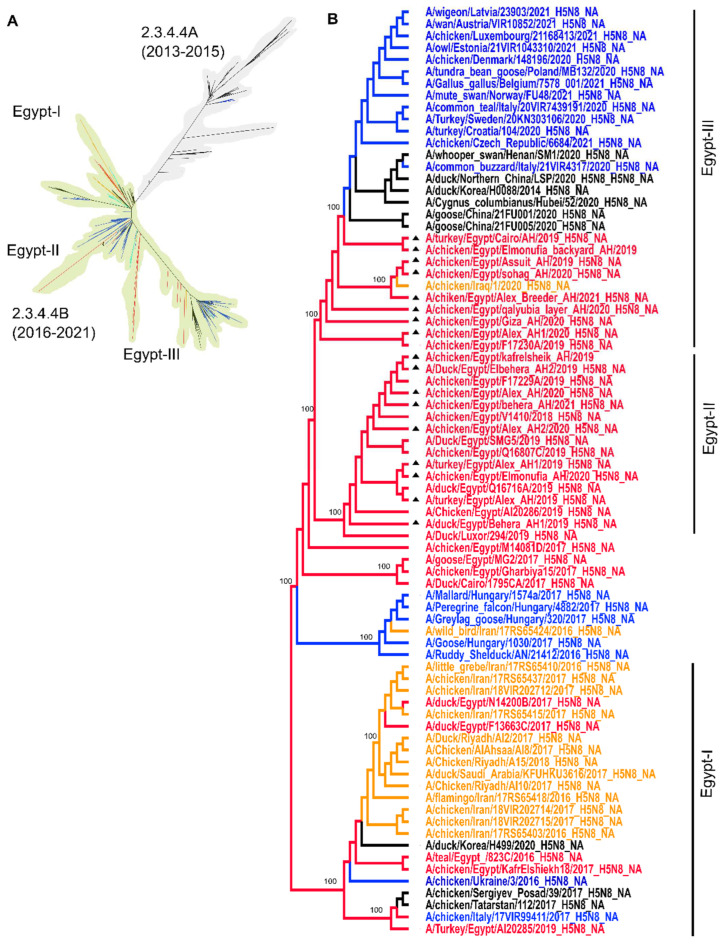
Phylogenetic relatedness of the NA of Egyptian H5N8 to Eurasian viruses. Nucleotide sequences of all-full or near-full NA (*n* = 1994) genes of H5N8 viruses from Asia, Africa, the Middle East and Europe were retrieved on 21 January 2022 and aligned using MAFFT and the tree was generated by IQTree (**A**). The phylogenetic tree of the Egyptian H5N8 viruses, including new sequences generated in this study (marked in black triangles), and selected sequences from other countries was generated by MrBayes implemented in Topali v.2 using the GTR + G model. Trees were generated after selecting 4 chains of 10,000,000 replicates and 25% buried-in parameters. Posterior probability values are shown on the main nodes (**B**). Egyptian H5N8 sequences are shown in red, H5N8 sequences from the Middle East are shown in orange, sequences from Europe are shown in blue and Asian viruses are in black. Branches depicted in cyan in panel A refer to sequences from other African countries.

The phylogroups of the NA of H5N8 in Egypt and the Middle East were relatively similar to those of the HA, although the available number of NA sequences was less than that of the HA (Figure 2A). Similar to the HA phylogeny, the NA sequences in phylogroup-I represent a few Egyptian viruses from wild birds and poultry in 2016–2019 along with viruses from the Middle East and Eurasia. Phylogroup-II contains only recent Egyptian viruses from 2019/2021, including nine new sequences generated in this study, clustered together representing the endemic strains in poultry. Phylogroup-III is a daughter cluster to phylogroup-II and is formed of Egyptian H5N8 viruses from 2019/2021 including eight new sequences generated in this study and viruses from Eurasia and a contemporary virus from chickens in Iraq (Figure 2B).

### 3.4. Comparison of HA Protein with Commercially Licensed H5 Vaccines in Poultry in Egypt

We compared the identity and number of amino acid differences in the HA of Egyptian viruses and commercially available vaccines in Egypt (Table 2). The results showed that the Egyptian H5N8 viruses share a low genetic identity (75.3–92.0%) compared to vaccines based on the historic H5N2 viruses from Potsdam/1986 and Mexico/1994 (Table 2). In contrast to the H5N1 viruses in 2006–2008, these vaccines are not commonly used to protect against the current H5N8 viruses in poultry in Egypt. Conversely, a higher genetic identity was observed compared to clade 2.2 (88.7–96.4%). The highest identity was observed with clade 2.3.4.4 from China (Re8) and wild birds in Egypt in 2016 (EGY16/H5N8) (94.9–100%) (Table 2) and therefore they are preferred in the field over other vaccines.

**Figure 3:**
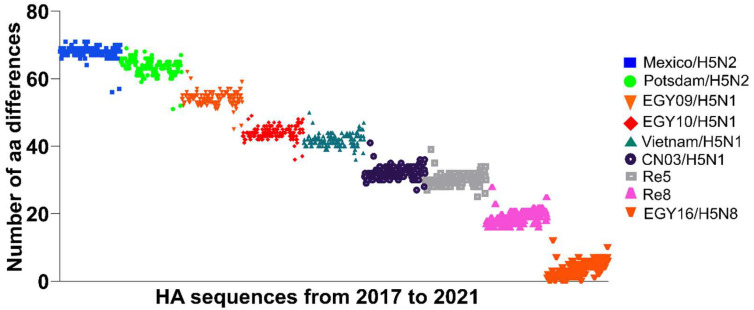
Amino acid differences of Egyptian H5N8 from 2017 to 2021 and licensed H5 vaccine strains in poultry in Egypt. Amino acid sequences of Egyptian H5N8 from 2017 to 2021 were retrieved from GISAID. All sequences including those generated in this study were aligned against different vaccine strains. Number of amino acid differences compared to the vaccine strain. Number of amino acid (aa) differences are shown in the y-axis. Each dot represents one HA sequence and sequences are arranged from 2017 to 2021. The figure was generated by GraphPad Prism 9.0.0 and was further edited using Inkscape 0.92. For the abbreviations of the vaccine strains, refer to Table 2.

The identity was calculated for the mature HA protein after the sequence of the signal peptide. For Mexican H5N2, only HA1 sequence is available.Nevertheless, our analysis indicated a temporal trend for the increased number of aa differences, particularly against clade 2.3.4.4 based vaccines (Figure 3).

**Figure 4:**
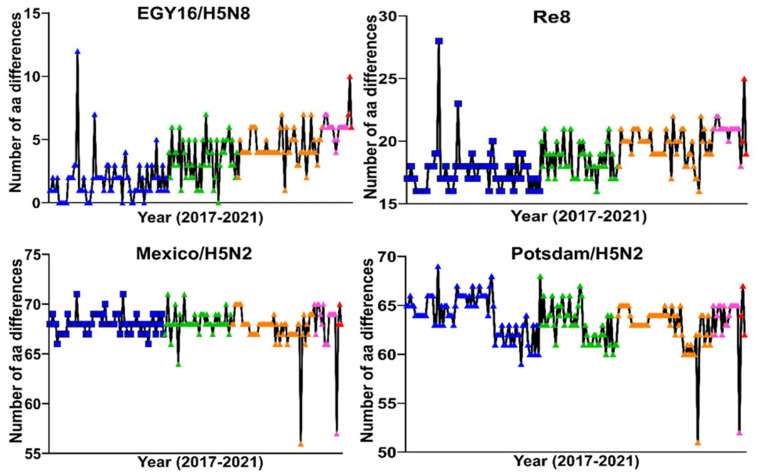
Number of amino acid differences of Egyptian H5N8 from 2017 to 2021 compared to selected vaccine strains. HA sequences of viruses isolated from 2017 (blue), 2018 (green), 2019 (orange), 2020 (pink) and 2021 (red) were compared to vaccine strains. Clade 2.3.4.4 EGY16/H5N8 (upper left) and Re8 (upper right) are commonly used in poultry in Egypt, while historic H5N2 virus-based vaccines (lower panels) are less used. Although clade 2.3.4.4 viruses are closely related to the Egyptian viruses, there is an increasing number of aa differences in the Egyptian H5N8 viruses particularly from 2020/2021 compared to the H5N2 vaccines. For the abbreviations of the vaccine strains, refer to Table 2.

The Egyptian H5N8 viruses isolated in 2020/2021 possessed a higher number of aa differences compared to their ancestors in comparison to clade 2.3.4.4 vaccines, indicating a continuous genetic drift from the vaccine strains (Figure 4).

Compared to the vaccine strain from the local Egyptian H5N8 strain (A/green-winged teal/Egypt/877/2016), the HA protein of the new viruses generated in this study in phylogroup Egypt-II possessed R72N/S (*n* = 9/9) in addition to S94R (*n* = 4/9), R169Q (*n* = 4/9), T188I (*n* = 4/9), V522A (*n* = 2/9) and V532M (*n* = 2/9) (Table 3). Similarly, new viruses generated in this study in phylogroup Egypt-III possessed T140A (*n* = 10/11), N236D (*n* = 8/11), V522A (*n* = 11/11) and V532M (*n* = 10/11). R169Q and T188I were rarely observed in this group (Table 3). All Egyptian viruses generated in this study possessed E268G. These mutations were also enriched in comparison to other vaccines, e.g., Re8 vaccine (data not shown). We further analyzed the prevalence of these mutations in the European H5N8 viruses from 2017 to 2021 (*n* = 1169) (Supplementary Table S1). The prevalence of N/S72, R94, Q169 and I188 in these viruses was ≤0.6% and a higher prevalence for A140 (48.2%), D236 (48.1%), G268 (93.7%), A522 (48.3%) and M532 (50.2%) was observed. These mutations are predicted to be in (residues 72, 140) or adjacent to (residues 94, 169, 188) the HA immunogenic epitopes [39] (Supplementary Table S1).

## 4. Discussion

The panzootic HPAIV H5N8 clade 2.3.4.4 devastated the poultry industry in Asia, Europe, North America and Africa [4,5]. Therefore, vaccination against H5N8 has been successfully implemented in several countries to limit the tremendous economic losses in the poultry industry [40,41]. The vaccination of poultry against HPAIV is highly useful to reduce morbidity, mortality, virus excretion and bird-to-bird transmission. However, mismatched vaccines or improper vaccination may accelerate virus evolution and may lead to the escape from vaccine-induced antibodies [19,32,42]. In China, the regular update of AIV vaccines was efficiently successful at mitigating the economic losses in poultry and reducing the public health threat caused by AIV [11,16,17,40]. Outside China, little is known about the prevalence and genetic properties of H5N8 viruses in vaccinated poultry. An experimental study described the inefficiency of non-clade 2.3.4.4 H5 vaccines to prevent morbidity, mortality or shedding in chickens that were experimentally infected with an Egyptian H5N8 virus isolated from wild birds in 2016 [41]. However, the prevalence of HPAIV H5N8 in vaccinated flocks in Egypt is largely unknown, which has been described in this short communication paper.

Our surveillance showed a high prevalence rate of H5N8 in vaccinated flocks, which received the vaccine up to four times. Vaccinal breaks can be attributed to factors related to the vaccination process (e.g., improper vaccination, vaccination coverage), the bird (e.g., age, species, immune suppression) or the vaccine strain (e.g., seed virus, antigen mass, storage and transport conditions) [42]. One of the major limitations of our passive surveillance is the lack of data on the levels of antibodies after vaccination or the cross-reactivity between the viruses isolated in this study and vaccine-induced antibodies. However, the high prevalence of AIV H5N8 in vaccinated poultry, the increased distinction of circulating 2020/2021 viruses from vaccine strains and the selection of mutations in the HA immunogenic epitopes (i.e., R72N/S, T140A) highlight the need to revise the efficiency of the currently licensed H5 vaccines in Egypt. Furthermore, our sequence analyses confirmed previous findings of multiple introductions of H5N8 from Eurasia into Egypt via wild birds in 2016–2019 [23,24,25,36,43] and revealed the establishment of a new sub-cluster in 2020/2021. These results indicate the importance of poultry in Egypt in the global epidemiology of H5N8 clade 2.3.4.4b along the migration flyways and as an endemic hotspot for H5N8 in the Middle East. The impact of mutations in viruses of phylogroups Egypt-II and Egypt-III compared to the extinct phylogroup Egypt-I on biological fitness remains to be investigated. Last but not least, among the reasons for the endemicity of H5N1 clade 2.2.1 in poultry in Egypt since 2006 were the poor biosecurity measures, low vaccination coverage, improper vaccination, the use of outdated vaccine strains (e.g., H5N2 from 1973), the absence of an efficient monitoring system, inadequate training of field technicians and the lack of periodical evaluation and updating of AIV vaccines [30,31,44]. To control H5N8 in poultry in Egypt, these factors should be seriously considered.

Together, HPAIV H5N8 was detected in 45.1% of H5-vaccinated poultry flocks in Egypt in 2019/2021. The sequence analysis indicated three phylogroups representing endemic H5N8 viruses and multiple introductions into Egyptian poultry from Eurasia. The efficacy of current vaccines should be evaluated and biosecurity measures should be improved. Poultry in Egypt is vulnerable to the frequent introduction of H5N8 viruses from Eurasia and a hotspot for H5N8 in the Middle East.

## Figures and Tables

**Table 1:** H5N8 viruses isolated in this study.

Virus	Date	Locality	Breed	Age	Number of Animals	Frequency of Vaccination	Accession Numbers	
							HA	NA
A/duck/Egypt/Behera-AH1/2019	October 2019	Elbehera	Broiler	34d	18,000	Once	EPI1999284	EPI1999283
A/turkey/Egypt/Alex-AH/2019	October 2019	Alexandria	Turkey	55d	6000	Once	EPI1999276	EPI1999275
A/chicken/Egypt/Assuit-AH/2019	November 2019	Assuit	Broiler	28d	10,000	Not	EPI1999303	EPI1999302
A/chicken/Egypt/Kafrelsheik-AH/2019	November 2019	Kafrelsheik	Broiler	29d	20,000	Once	EPI1999290	EPI1999289
A/chicken/Egypt/Elmonufia-backyard-AH/2019	December 2019	Elmonufia	Local Breed	44d	500	Not	EPI1999296	EPI1999295
A/turkey/Egypt/Cairo/AH/2019	December 2019	Cairo	Turkey	60d	7000	Once	EPI1999274	EPI1999273
A/turkey/Egypt/Alex-AH1/2019	December 2019	Alexandria	Turkey	90d	5000	Once	EPI1999279	EPI1999277
A/duck/Egypt/Elbehera-AH2/2019	December 2019	Elbehera	Duck	40d	10,000	Once	EPI1999281	EPI1999280
A/chicken/Egypt/Cairo-HC11B-AH/2020	January 2020	Cairo	Broiler	33d	21,000	Once	EPI1999299	n.d.
A/duck/Egypt/Behera-HB2-AH/2020	January 2020	Elbehera	Duck	45d	12,000	Once	EPI1999282	n.d.
A/chicken/Egypt/Giza-AH/2020	January 2020	Giza	Layer	24 wks	20,000	3 times	EPI1999294	EPI1999293
A/chicken/Egypt/Alex-AH/2020	January 2020	Alexandria	Broiler	31d	17,000	Once	EPI1999308	EPI1999306
A/chicken/Egypt/Elmonufia-AH/2020	February 2020	Elmonufia	Broiler	26d	18,000	Once	EPI1999298	EPI1999297
A/chicken/Egypt/Giza-HG4L-AH/2020	February 2020	Giza	Broiler	28d	16,000	Once	EPI1999292	n.d.
A/chicken/Egypt/Sohag-AH/2020	February 2020	Sohag	Broiler	32d	15,000	Not	EPI1999286	EPI1999285
A/chicken/Egypt/Qalyubia-layer-AH/2020	March 2020	Qalyubia	Layer	32 wks	35,000	3 times	EPI1999288	EPI1999287
A/chicken/Egypt/Alex-AH2/2020	March 2020	Alexandria	Broiler	19d	22,000	Once	EPI1999310	EPI1999309
A/chicken/Egypt/Alex-AH1/2020	May 2020	Alexandria	Broiler	29d	15,000	Once	EPI1999312	EPI1999311
A/chicken/Egypt/Behera-AH/2021	February 2021	Elbehera	Broiler	33	12,000	Once	EPI1999301	EPI1999300
A/chicken/Egypt/Alex-Breeder-AH/2021	March 2021	Alexandria	Broiler Breeder	40 wks	16,000	4 times	EPI1999305	EPI1999304

Age: d = day, wks = weeks. n.d. = not done.

**Table 2:** Genetic identity of Egyptian H5N8 viruses to commercially available H5 vaccines currently used in poultry in Egypt.

No.	Vaccine Seed Virus	Subtype	Abbreviation	Clade/Lineage	Accession Numbers (aa)	Company	AA Identity to Egyptian H5N8 (Min–Max)
1	A/chicken/Mexico/232/1994	H5N2	Mexico/H5N2	North American	AAR88841	Ceva, Mexico	75.3–90.1
2	A/duck/Potsdam/1402-6/1986	H5N2	Potsdam/H5N2	Eurasian	ABI84497	Intervet, The Netherlands	87.2–92.0
3	A/chicken/Egypt/18-H/2009	H5N1	EGY09/H5N1	2.2.1.1	ADG28676	Harbin Veterinary Research Institute, China	88.7–94.2
4	A/duck/Egypt/M2583D /2010	H5N1	EGY10/H5N1	2.2.1.1	AEP37317	ME-VAC, Egypt	90.8–96.4
5	A/chicken/Vietnam/C58/2004	H5N1	Vietnam/H5N1	1	AAW80718.1	Zoetis, USA	90.9–96.7
6	A/duck/China/E319-2/2003	H5N1	CN03/H5N1	2.3.2	AAR99628	Boehringer Ingelheim, Germany	92.5–98.4
7	A/duck/Anhui/1/2006	H5N1	Re5	2.3.4	ADG59091	QYH, China	92.9–98.4
8	A/chicken/Guizhou/4/2013	H5N1	Re8	2.3.4.4	EPI675769	Merial, USA & QYH, China	94.9–97.1
9	A/green-winged teal/Egypt/877/2016	H5N8	EGY16/H5N8	2.3.4.4b	ART29489	ME-VAC, Egypt	97.8–100

**Table 3:** Amino acid differences in Egyptian H5N8 viruses compared to the vaccines strain.

Phylogroup	Virus/aa Position (H5 Numbering) *	72	94	140	169	188	236	268	522	532
Vaccine strain	A/green-winged teal/Egypt/877/2016	R	S	T	R	T	N	E	V	V
Egypt-II	A/Duck/Egypt/Elbehera_AH2/2019	N	.	.	.	.	.	G	.	.
	A/chicken/Egypt/kafrelsheik_AH/2019	N	.	.	.	I	.	G	.	.
	A/chicken/Egypt/Alex_AH1/2020	N	.	.	.	I	.	G	A	.
	A/chicken/Egypt/Alex_AH/2020	N	.	.	.	I	.	G	A	M
	A/chicken/Egypt/Alex_AH2/2020	N	.	.	.	I	.	G	.	.
	A/chicken/Egypt/Elmonufia_AH/2020	S	R	.	Q	.	.	G	.	M
	A/turkey/Egypt/Cairo/AH/2019	S	R	.	Q	.	.	G	.	.
	A/turkey/Egypt/Alex_AH/2019	S	R	.	Q	.	.	G	.	.
	A/duck/Egypt/Behera_AH1/2019	S	R	.	Q	.	.	G	.	.
Egypt-III	A/chicken/Egypt/behera-AH/2021	.	.	A	Q	.	.	G	A	.
	A/chiken/Egypt/Alex-Breeder-AH/2021	.	.	A	Q	.	.	G	A	M
	A/chicken/Egypt/Giza-AH/2020	.	.	A	.	.	D	G	A	M
	A/chicken/Egypt/Elmonufia-backyard-AH/2019	.	.	A	.	.	D	G	A	M
	A/chicken/Egypt/qalyubia-layer-AH/2020	.	.	A	.	N	D	G	A	M
	A/chicken/Egypt/sohag-AH/2020	.	.	A	.	.	D	G	A	M
	A/chicken/Egypt/Assuit-AH/2019	.	.	A	.	.	D	G	A	M
	A/Duck/Egypt/Behera-HB2-AH/2020	.	.	A	.	.	D	G	A	M
	A/Chicken/Egypt/Giza-HG4L-AH/2020	.	.	.	.	.	D	G	A	M
	A/Chicken/Egypt/Cairo-HC11B-AH/20	.	.	A	.	.	D	G	A	M
	A/turkey/Egypt/Alex-AH1/2019	.	.	A	.	.	.	G	A	M

* Residue numbering is based on the sequence of the mature H5 HA protein. Dots indicate residues identical to the vaccine strain.

## Data Availability

Sequences generated in this study are deposited in the GISAID and assigned accession numbers: EPI1999273 to EPI1999312.

## Supplementary Materials

The following supporting information can be downloaded at: https://www.mdpi.com/article/10.3390/v14071431/s1, Table S1: Prevalence of HA mutations in the European H5N8 viruses from 2017 to 2021.
