# Supplementary material for: Isolation of Genetically Diverse H5N8 Avian Influenza Viruses in Poultry in Egypt, 2019–2021
Source: Viruses. 2022 Jun 29;14(7):1431. doi: 10.3390/v14071431 (PMC9320977; doi:10.3390/v14071431)
Supplement: Supplementary file 1 [file viruses-14-01431-s001.zip › viruses-1764755-supplementary.pdf]

Supplementary Table S1. Prevalence of HA mutations in the European H5N8 viruses from 2017 to 2021.

Virus / aa Position (H5 Numbering)	Vaccine Strain A/Green-Winged Teal/Egypt/877/2016 (H5N8)	European H5N8 (2017–2021) n= 1169	Predicted HA Domain or Structure
72	R	R (99.4%), S (0.6%)	Immunogenic epitope E
94	S	S (99.6%), R (0.0%)	Adjacent to immunogenic epitope E
140	T	T (51.1%), A (48.2%)	Immunogenic epitope B
169	R	R (99.5%), Q (0.3%)	Adjacent to immunogenic epitope B
188	T	T (99.9%), I (0.1%)	Adjacent to immunogenic epitope D and receptor binding sites
236	N	N (51.9%), D (48.1%)	
268	E	E (6.3%), G (93.7%)	
522	V	V (51.7%), A (48.3%)	Transmembrane domain
532	V	V (49.3%), M (50.2%)	Transmembrane domain

Sequences of European H5N8 viruses from 01.01.2017 to 31.12.2021 (n= 1319) were retrieved from GISAID and aligned using MAFFT. The retrieval date was 17-05-2022. HA proteins with duplicate entries and with incomplete or ambiguous sequences were removed from the analysis. A total of 1169 full or near full HA sequences of European H5N8 viruses were analysed using Geneious Software. The prevalence rate of mutations enriched in the Egyptian H5N8 viruses compared to the vaccine strain was calculated. .
